# Modularity of gene-regulatory networks revealed in sea-star development

**DOI:** 10.1186/1741-7007-9-6

**Published:** 2011-01-31

**Authors:** Carmel McDougall, Bernard M Degnan

**Affiliations:** 1 1Centre for Marine Science, School of Biological Sciences, University of Queensland, Brisbane 4072, Australia

## Abstract

Evidence that conserved developmental gene-regulatory networks can change as a unit during deutersostome evolution emerges from a study published in *BMC Biology*. This shows that genes consistently expressed in anterior brain patterning in hemichordates and chordates are expressed in a similar spatial pattern in another deuterostome, an asteroid echinoderm (sea star), but in a completely different developmental context (the animal-vegetal axis). This observation has implications for hypotheses on the type of development present in the deuterostome common ancestor.

See research article: http://www.biomedcentral.com/1741-7007/8/143/abstract

## 

The deuterostomes comprise a group of related phyla that includes the echinoderms (sea urchins, sea stars and their relatives), the hemichordates (for example, acorn worms and pterobranchs) and the chordates (the phylum to which vertebrates belong). Understanding the origin and evolution of deuterostome phyla (Figure 1) is hampered by the vastly different developmental modes and adult body plans exhibited by their members. Deuterostomes can either develop indirectly into the juvenile/adult form via one or more larval phases, or develop directly, lacking a morphologically distinct larval stage. Within the indirect developers, larvae can be either planktotrophic, actively feeding and possessing a functional larval gut, or lecithotrophic, gaining nourishment from yolk reserves in the egg. In the latter case, the gut often forms during metamorphosis.

**Figure 1:**
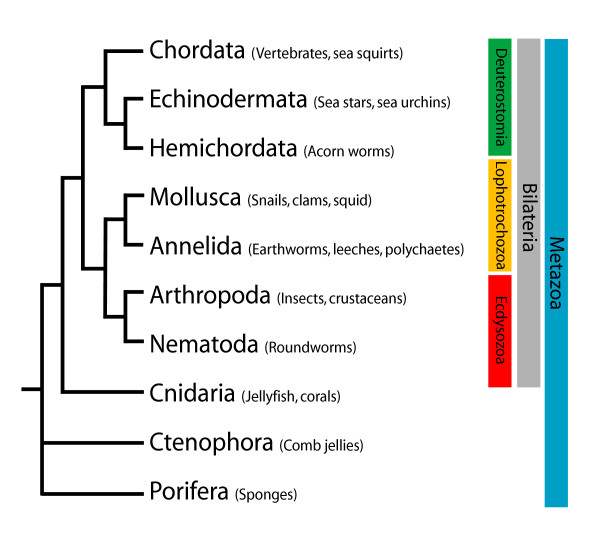
**The position of the deuterostomes within the Metazoa**.

## Deployment of a developmental gene-regulatory network in different deuterostomes

The presence of a direct-developing deuterostome ancestor has been suggested on the basis of evidence that includes the discovery of similar molecular pathways responsible for patterning of prospective neural tissue (the neuroectoderm) along the anterior-posterior (AP, head to tail) axis of direct-developing hemichordates and vertebrates (which both have bilateral symmetry) [1]. In a recent paper in *BMC Biology*, however, Yankura and colleagues [2] now find that the indirectly developing sea star *Patiria miniata *(which has radial symmetry as an adult) also uses components of these pathways in spatially restricted patterns, but in this case to pattern the larval ectoderm along the animal-vegetal (AV) axis (the axis running from the animal pole to the vegetal pole of the early embryo or blastula).

This finding indicates that the genes involved in these patterning processes could be components of a conserved gene regulatory network (GRN) - a set of genes that operate together in a predicted pattern of activation or repression to control a particular process within an organism - and that this GRN is deployed regardless of developmental mode. The sea-star data reveal that this GRN is not restricted to patterning the anterior neuroectoderm or brain. The corollary to this is that the presence of conserved GRNs cannot by itself be taken as evidence for the particular form or developmental mode of a common deuterostome ancestor. Yankura *et al. *[2] also describe the uncoupling of a conserved retinal determination GRN from the anterior brain GRN, both of which are expressed in the neuroectoderm of chordates. In both sea stars and sea urchins, however, the anterior brain GRN is expressed in the larval ectoderm, as noted earlier, while the 'retinal' GRN is expressed in the larval mesoderm. Their study therefore highlights the evolvability of GRNs in deuterostome development.

## GRNs as phylogenetic characters

Evolutionary speculation has traditionally been based on morphological studies. But as gene-expression data rolls in from an ever-increasing number of animal phyla, new insights and new questions arise. For example, the neuroectoderm of the annelid *Platynereis dumerilii *(a non-deuterostome) exhibits similar patterns of gene expression to various domains of the vertebrate neural tube [3], and eye formation in the fruit fly *Drosophila *(another non-deuterostome) and vertebrates utilizes similar regulatory pathways [4]. These highly conserved gene-expression patterns point to widespread conservation of GRNs across the animal kingdom. Changes in the way in which these networks are deployed might therefore provide phylogenetic signals that could aid our understanding of evolutionary processes.

Such evolutionary insights are already being revealed. One much debated evolutionary question concerns the origins of the larval stages of marine invertebrates (both deuterostome and non-deuterostome). Did the animal forms we see today evolve from a larva-like animal, or have extant larval types evolved multiple times independently, or is the biphasic (larval to adult) life cycle an ancient, shared feature of the Metazoa [5,6]? This issue is complicated by the huge diversity of larval forms present today, and uncertainty over the homology of various larval structures.

Many marine invertebrate larvae, from different phyla, have a tuft of cilia at their apical (anterior) end (the apical tuft), which is generally thought to have a sensory function. Whether this apical tuft is homologous between the different larval types or arose multiple times during evolution is unknown, and weighs in on the debate of possible independent origins of marine larvae. Expression of the transcription-factor gene *COE *in the vicinity of apical tufts of cnidarian, mollusc, annelid and echinoderm larvae suggests underlying genetic similarities and possible homology of these structures [7]. However, when Dunn and colleagues [8] examined the expression of genes involved in cilium formation in the apical ectoderm of an echinoderm and a mollusc, they found that although these genes are conserved, the upstream genes that regulate the network are different in the two species. They conclude that the apical tuft is not homologous between molluscs and echinoderms, which is consistent with multiple origins of ciliated larval types.

## Modularity of GRNs and their redeployment in evolution

The study of Dunn *et al. *[8] also demonstrates that GRNs can drive evolution via the linking of existing GRNs to different inputs. It is therefore becoming evident that the redeployment of GRNs at different times of development, in different developmental contexts, or in completely new territories within the organism, is an important process in metazoan evolution, and that the circuitry of these networks can provide more robust evolutionary information than the expression patterns of individual genes.

Historically, the notion that large-scale evolution could be driven by a multiplicity of small changes in DNA sequences was theoretically challenging, as the majority of these changes are likely to be deleterious, and cause decreased fitness. The discovery of GRNs makes this conceptually less of a problem, as base-pair changes in the regulatory region of one gene could alter the binding affinities of various transcription factors, effectively placing the entire network under the operation of different drivers. One can then envisage the situation where a given gene network could be induced to turn on at a different stage of development (a heterochronic shift) or in a different spatial location within the organism. This has several implications, namely that large-scale morphological change could occur quickly, and that similar rearrangements could occur by chance in different contexts.

In many cases, the conservation of a number of gene-expression patterns in a similar process in two organisms (usually in two different phyla) is used as evidence that a shared GRN is in operation, and that, presumably, this GRN was used in the same way in the last common ancestor of those two organisms. Even if this is the case, it is generally not appreciated that a particular GRN can also be deployed in different ways among more closely related taxa. Across metazoans, it has been demonstrated that developmental mode is evolutionarily labile, with closely related species displaying a mixture of planktotrophy, lecithotrophy and direct development (for example, in gastropods [9] and echinoderms [10]). The development of body plans in animals with different life-history strategies requires heterochronic shifts in the deployment of GRNs [10]. Therefore, the existence of common mechanisms of patterning in distantly related animals with similar life strategies does not necessarily provide iron-clad evidence for the presence of that developmental type in their last common ancestor. Indeed, the regulatory network similarities observed by Yankura *et al*. [2] between head/anterior brain patterning genes in the bilaterian hemichordates and vertebrates and their orthologs in the sea star ectoderm reveals a deeply conserved systemic patterning system that may antedate the bilaterian morphogenetic systems with which it is often associated (that is, the axial neuroectoderm). Such observations should be factored into reconstructions of the origin and early evolution of the deuterostomes, which will require the analysis of contemporary echinoderms, hemichordates and chordates with both directly and indirectly forming body plans.

An exciting and necessary direction of future research will involve understanding the mechanisms by which heterochronic shifts in development are controlled. For example, how is the switch between planktotrophy and lecithotrophy governed? A key facet of this research is the question of the inputs that govern the switching of key GRNs and how these operate in the context of different developmental modes. Closely related species with varying life-history strategies, such as sea urchins of the genus *Heliocidaris *[10], are ideal systems in which to investigate these questions. Such research will also contribute to the general understanding of the modular nature of GRNs and their underlying role in the major changes in developmental programs observed throughout the Metazoa.

By investigating gene expression in the AV axis of an indirectly developing sea star, Yankura *et al*. [2] provide further evidence for the modularity of GRNs and for their evolvability and differential deployment in related animals with different developmental modes. These comparisons and conclusions are facilitated by the diversity of model species and the extent of the genomic resources that are available for deuterostome species, making them the metazoan group currently best suited to unravel the function of GRNs in the evolution of animal body plans.
